# A story for Pepper and Nao: applying the learning by teaching paradigm in children with patterns of neuromotor impairment

**DOI:** 10.3389/fdgth.2026.1929794

**Published:** 2026-10-14

**Authors:** Benedetta Del Lucchese, Maria Chiara Di Lieto, Antea Scrocco, Elisa Matteucci, Laura Fiorini, Elena Pescini, Elena Beani, Annalisa Cotardo, Elena Vanni, Eugenia Conti, Valentina Menici, Federica Camuncoli, Gloria Mangani, Marco Vincenzo Maselli, Elena Adelucci, Jasmine Pani, Filippo Cavallo, Chiara Pecini, Eleonora Diella, Fabio Storm, Emilia Biffi, Mattia Chiappini, Sabrina Signorini, Roberta Nicotra, Consortium Fit4Med-Pepper Storie, Giuseppina Sgandurra

**Affiliations:** 1 Department of Developmental Neuroscience, IRCCS Fondazione Stella Maris, Pisa, Italy; 2 Department of Industrial Engineering, University of Florence, Florence, Italy; 3 Department of Clinical and Experimental Medicine, University of Pisa, Pisa, Italy; 4 Department of Education, Languages, Interculture, Literature and Psychology (FORLILPSI), University of Florence, Florence, Italy; 5 Scientific Institute, IRCCS Eugenio Medea, Bosisio Parini, Lecco, Italy; 6 Department of Psychology, University of Torino, Torino, Italy; 7 Department of Child Neurology and Psychiatry, IRCCS Mondino Foundation, Pavia, Italy; 8 Department of Brain and Behavioral Sciences, University of Pavia, Pavia, Italy

**Keywords:** children, engagement, executive functions, learning by teaching, neuromotor impairment, social robot

## Abstract

**Background:**

Social robots are increasingly used in educational and clinical contexts to enhance engagement and learning, particularly in children with neurological and neurodevelopmental disorders. The Learning by Teaching paradigm, in which children actively teach social robots, promotes cognitive and metacognitive processes such as planning, self-efficacy, and executive functioning; however, controlled and systematic evidence supporting its effectiveness remains limited. Children with neuromotor disorders frequently present with closely intertwined motor and cognitive planning difficulties, which significantly impact learning and everyday functioning. In this context, social robot–mediated interventions based on the Learning by Teaching paradigm may represent a promising approach. The current multicenter randomized controlled trial (RCT) explores the feasibility and effectiveness of this approach to enhance cognitive and motor planning in children with neuromotor impairments, and to examine potential generalization effects on other cognitive domains and engagement.

**Methods:**

The main aim of this RCT study is to evaluate the feasibility and effectiveness of a social robot-mediated Learning by Teaching intervention in children aged 7–16 years with neuromotor impairments. Eighty participants will be randomly assigned to an Experimental Group (EG) receiving social robot training or to a Control Group (CG) continuing standard care. The intervention consists of 20–25 sessions in which children teach a humanoid social robot (Pepper or NAO) to order picture-based stories of increasing complexity. Assessments will be performed at baseline (T0), post-intervention or control period (T1), and follow-up/post-treatment (T2). Primary outcomes include planning abilities assessed through picture arrangement and an *ad hoc* motor planning task. Secondary outcomes encompass executive functions, visuo-spatial memory, attention, inhibition, narrative production, visual-perceptual skills, and reading comprehension. Engagement will be measured using a multimodal approach integrating behavioral, cognitive, emotional, and physiological indicators derived from video, audio, and wearable sensors.

**Results:**

The study will examine improvements in planning and motor programming abilities and potential generalization effects on executive functions and reading comprehension. Additionally, the predictive and mediating role of engagement in intervention effectiveness will be reported.

**Conclusions:**

This research protocol proposes an innovative, engagement-focused rehabilitation approach integrating cognitive, motor, and narrative processes through social robotics. Findings are expected to inform the development of evidence-based, personalized interventions for children with neuromotor disorders and to advance methodological approaches for assessing engagement in human–robot interaction.

**Clinical Trial Registration:**

ClinicalTrials.gov, identifier NCT06797050.

## 1 Introduction

In recent years, the use of social robots has become increasingly widespread in both educational and clinical settings, with applications involving children with typical development and as a promising tool for children with disabilities (1–4). Several studies conducted in school settings have shown that the increased involvement of children resulting from the presence of a social robot facilitates the learning phase, leading to better school performance (5–7). Increasingly, the literature has highlighted how the presence of a social robot can positively influence attention, satisfaction, and engagement in activities proposed to children (8) (9). Engagement in social robotics remains a complex and debated construct. Multiple indicators of the main components of engagement—emotional, behavioral, and cognitive—have been identified and should be considered when analyzing and studying interaction with robots (10–12). The emotional dimension concerns the user’s affective response—such as joy, satisfaction, or reduced anxiety (13) (14) the cognitive dimension involves attention, motivation, and active participation (15) (16) whereas the behavioral dimension includes proactivity, adaptability, and effort in maintaining interaction (15). Preliminary evidence examined engagement levels in relation to the robot’s anthropomorphic characteristics (both objective and perceived) (17) in this case, engagement was defined in relation to user experience and includes features of aesthetic pleasantness as well as the ability to maintain interaction with the social robot. According to the literature the robot’s anthropomorphic characteristics play a fundamental role in conveying emotional content during the interaction, fostering a greater engagement in the child (18). Nonetheless, the growing interest in measuring engagement presents significant methodological limitations, as current approaches are often based on small samples and non-standardized measures, preventing the identification of explanatory models for the effects of interaction and engagement with the robot.

An innovative and encouraging use of social robots, based on preliminary findings in school setting, is the application of the Learning by Teaching (LbT) paradigm, in which children are asked to teach another agent rather than “learn from” it, thereby supporting their own learning. “Learning by teaching” promotes self-efficacy and motivation, as well as the ability to take another’s perspective (theory of mind), since it requires adapting content and competencies according to the learner’s characteristics (19) (20). Using this paradigm with a social robot places the child in the position of adopting the learner’s point of view. In an educational setting, the social robot can be pre-programmed to provide specific prompts and responses, thus guiding the learning process while supporting engagement during the task, with potential mediating effects on motivational and cognitive aspects (21) and possible positive outcomes in terms of satisfaction and task performance (22).

Regarding the processes that can be trained through a Learning by Teaching paradigm with a social robot, particular emphasis has been placed on cognitive control and academic learning abilities (23) (20) (3). Cognitive control abilities, known as Executive Functions (EF), are higher-order control processes that enable the maintenance and manipulation of information (working memory), allow adaptation of strategies and behavior to changing demands (cognitive flexibility), and support the suppression of prepotent or irrelevant responses (inhibitory control). These capacities emerge from the integration of multiple processes and enable the planning and execution of goal-directed behavior while processing complex stimuli (24). These abilities play a central role in several aspects of daily life, underlying many other skills, including adaptive, social, academic, and motor domains. Although this paradigm appears promising and potentially very useful in rehabilitation, the available evidence is still scarce and mainly limited to single-case reports (e.g., 3), substantially restricting the generalizability of the findings. Further research employing more rigorous methodologies and larger samples is therefore needed to better ascertain LbT paradigm effectiveness in rehabilitative contexts.

In neuromotor disorders, despite the wide heterogeneity of etiological and clinical characteristics, the impairments often extend beyond overt motor difficulties, encompassing neuropsychological processes and academic abilities. Particularly, vulnerabilities in planning processes are observed at both motor and cognitive levels, with severity varying partly as a function of the underlying clinical condition and task demands, as reported in children with neurofunctional impairments [e.g., Developmental Coordination Disorder (25),] or with congenital brain lesion [e.g., Cerebral Palsy (26)]. Motor planning can be defined as a set of operations supporting goal anticipation, selection and temporal organization of movement sequences, preparation for execution, and online monitoring of outcomes (27). It encompasses multiple components, including anticipatory control, preparation, motor programming, and motor imagery. Deficits in these mechanisms are associated with multiple levels of impairment in the execution of motor actions (28–32). Strongly associated with motor planning, EF difficulties are often present in children with neuromotor disorders, which can result in a compromised capacity to plan and organize actions, regulate attention, and manage complex cognitive tasks (24), thereby negatively impacting both the main motor impairment (33) and other related cognitive processes and academic skills (34). Among those, written text comprehension is particularly critical, as it constitutes a transversal and crucial skill underpinning learning across disciplines and supporting everyday functioning. This ability relies on complex, multidimensional processes requiring the integration of decoding skills, logical and inferential reasoning, cognitive control mechanisms (e.g., selective attention, inhibition, and working memory), and metacognitive abilities (e.g., monitoring, planning, and self-regulation) to build coherent mental representations of a text, even under conditions of ambiguity (35–37). Despite inconclusive evidence in the literature concerning academic achievement and written text comprehension, difficulties in these domains have been frequently documented in neuromotor disorders (38–41).

Given the frequent inter-relationships among neuromotor, executive function, and text processing impairments, there is a strong rationale for designing rehabilitation tasks that involve these domains concurrently. To this purpose, a picture sequencing task, which involves arranging images in the correct order to create a coherent story, has been developed for this study protocol. Besides being an ecological and enjoyable activity for the child, the picture sequencing task allows work on visual, spatial, sequential, and temporal processing of images, supporting multiple higher-order processes such as working memory, attention, reasoning, motor and cognitive planning (42) (43), as well as narrative organization skills underlying both comprehension and text production. Embedding this task within a LbT paradigm involving a social robot could amplify its rehabilitative impact on these processes, both through higher engagement elicited by the robot’s presence and through the child’s role as the robot’s teacher. Within this framework, the “A Story for Pepper and Nao” project, designed as an exploratory RCT, aims to assess the feasibility and then evaluate the effectiveness of a Learning by Teaching training paradigm implemented with a social robot through a picture sequencing task in children with neuromotor disorders. The training effectiveness will be evaluated through improvements in logical and inferential reasoning, and motor programming, as well as potential transfer effects to secondary non-targeted domains such as reading comprehension and motor skills, while concurrently investigating the predictive contribution of children’s behavioral, cognitive, and emotional engagement.

The study has been registered with ClinicalTrials.gov, number NCT06797050.

## 2 Materials and methods

### 2.1 Study design

This national, multicenter clinical study employs a waitlist randomized controlled design, with participants randomly allocated to either an Experimental or a Control group. Participant recruitment and the clinical trial will be conducted at the participating clinical centers (IRCCS Fondazione Stella Maris, Pisa; IRCCS Istituto Fondazione C. Mondino, Pavia; IRCCS Eugenio Medea—Associazione La Nostra Famiglia, Lecco), in collaboration with the University of Florence (Department of Industrial Engineering—DIEF and Department of Education, Languages, Interculture, Literatures and Psychology—FORLILPSI) which will support technological aspects, including robot programming, data analysis and engagement dynamics assessment.

After fulfilling the inclusion and exclusion criteria described below and providing signed informed consent, participants will undergo a feasibility assessment simulating a treatment session to verify the suitability of the task for each child. Eligible participants will then complete baseline evaluations (T0), including standardized neuropsychological, motor, and academic assessments. Participants will be then randomly allocated in a 1:1 ratio to either the Experimental Group (EG), which will receive social robot training, or to the Control Group (CG), which will continue with standard care. The random allocation sequence will be automatic generated independently at each participating center, and allocation will be implemented by the researchers responsible for participant enrollment at the respective center. No stratification factors were applied. After 3–4 months (T1), participants will be reassessed. For the EG, this evaluation will measure post-treatment changes, whereas for the CG, it will serve as a control time point to monitor any changes from baseline. Following T1, the CG will receive the same training as the EG, in line with the waitlist design. A final follow-up assessment (T2) will be conducted 3–4 months after T1, including standardized neuropsychological, motor, and academic tests. This assessment will serve as the follow-up for the EG and as the post-treatment evaluation for the CG, immediately after completing the training with the social robot (Figure 1).

**Figure 1:**
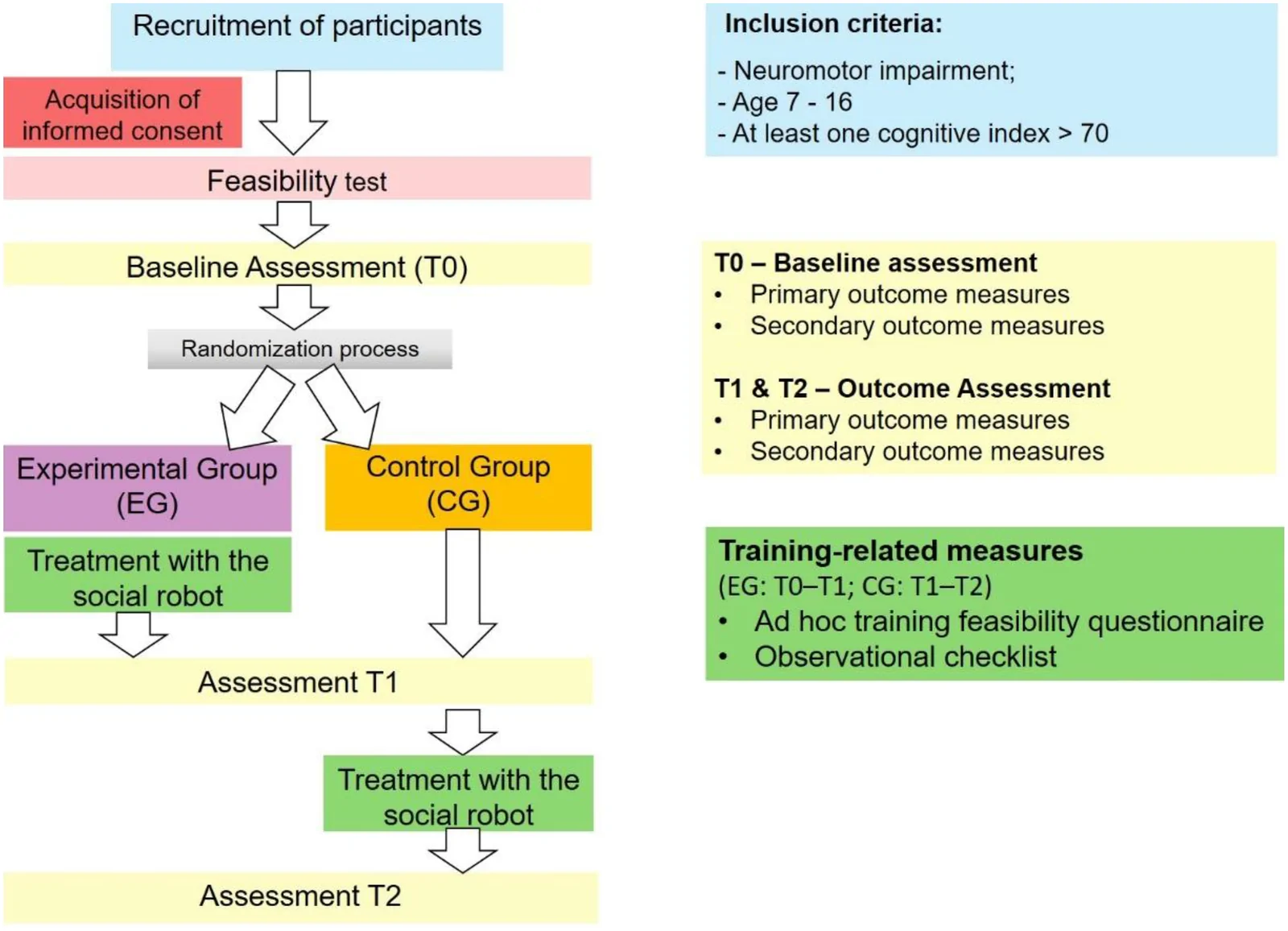
Study flowchart.

### 2.2 Participants

Participant recruitment will be conducted by the three clinical centers involved in the study (IRCCS Fondazione Stella Maris; IRCCS Eugenio Medea and IRCCS Fondazione C. Mondino), in accordance with predefined inclusion and exclusion criteria. Participants will be identified from existing databases of subjects who have previously consented to receive information and proposals for new research protocols, as well as among patients attending the above-mentioned structures for functional assessments and/or rehabilitation care. Participants will be recruited based on the following specific inclusion criteria:

- children diagnosed with a condition involving neuromotor aspects (e.g., Cerebral Palsy, Developmental Coordination Disorder, genetic syndromes, etc.);
- age between 7 and 16 years at the time of recruitment;
- presence of at least one cognitive index >70 on the WISC-IV or WISC-V, administered within the last year.

The following exclusion criteria will be considered:

- severe neurological, motor, or sensory deficit preventing participation in the training;
- psychiatric comorbidity that would preclude the feasibility of training.

Following invitation to participate in the study, eligible subjects and their families (i.e., parents or legal guardians) will receive the informed consent for participation and publication of results.

The research project was approved by the National Ethical Committee (n° 0159817 of 2024).

### 2.3 Sample size

*A priori* sample size of 77 participants was calculated using GPower (v3.1.9.4) based on a two-tailed paired-samples t-test, assuming an effect size of 0.375 (Cohen’s d), a significance level of 0.05, and a statistical power of 90%. The assumed effect size was selected based on previous evidence from pediatric cognitive/executive function interventions and social robot-mediated interventions, which reported small-to-moderate effects on related cognitive and behavioral outcomes (44, 45).

This calculation was used as an approximate reference for recruitment planning. However, because it was based on a paired-samples comparison, it does not directly correspond to the primary estimand of the present study—the between-group difference in change from baseline to post-intervention—and does not provide a formal estimate of the power to detect the group-by-time interaction. A design-consistent longitudinal power calculation would have required additional parameters, particularly the correlation between repeated measurements for the specific primary outcomes, which were not available from comparable studies.

To provide a limited margin for attrition and allow equal allocation between groups, the recruitment target was rounded to 80 participants. Children aged 7–16 years will be recruited across the three participating clinical centers and allocated to the Experimental Group (EG; *n* = 40) or the Control Group (CG; *n* = 40). Consequently, the planned sample size should not be interpreted as ensuring adequate power for the primary between-group comparison. Intervention effects will therefore be reported using effect estimates and 95% confidence intervals, with particular attention to their magnitude and precision. Participants will be recruited from among eligible children who completed the feasibility testing phase, as summarized in Figure 1.

### 2.4 Experimental training

During the training sessions, children in the EG will be asked to assist the social robot in organizing sequences of images depicting stories. The goal is to create a coherent narrative respecting temporal, causal, or inferential relationships.

The training task was developed through a collaborative effort between IRCCS Fondazione Stella Maris and the University of Florence. Based on the Learning by Teaching paradigm, the robot engages the participant by asking for assistance in reordering a sequence of picture-based stories. The story images are displayed on a dedicated user interface in a random order, and the participant is instructed to rearrange them into the correct narrative sequence (Figure 2). The stories are presented on the social robot’s tablet with gradually increasing complexity. Complexity increases along three dimensions: the number of images (from 3 to 7), the level of logical reasoning required (progressing from temporal to causal and inferential reasoning), and the number of characters and locations involved (from one to multiple). The clinical team of IRCCS Fondazione Stella Maris, composed of motor therapists and psychologists, developed a series of stories specifically designed for the rehabilitation program. A narrative script was created, comprising stories developed according to inclusive criteria and featuring characters from diverse ethnic backgrounds and with different types of disabilities. The stories focus on everyday life activities, including tasks that require specific motor sequences. The script was then graphically produced by professional designers under clinical supervision. A verbal description is also provided for each picture, which the child can listen again on demand as needed, thus providing additional support when reordering the story. Once the story has been reordered, the children can also listen to the entire sequence verbally before deciding whether to confirm their selection.

**Figure 2:**
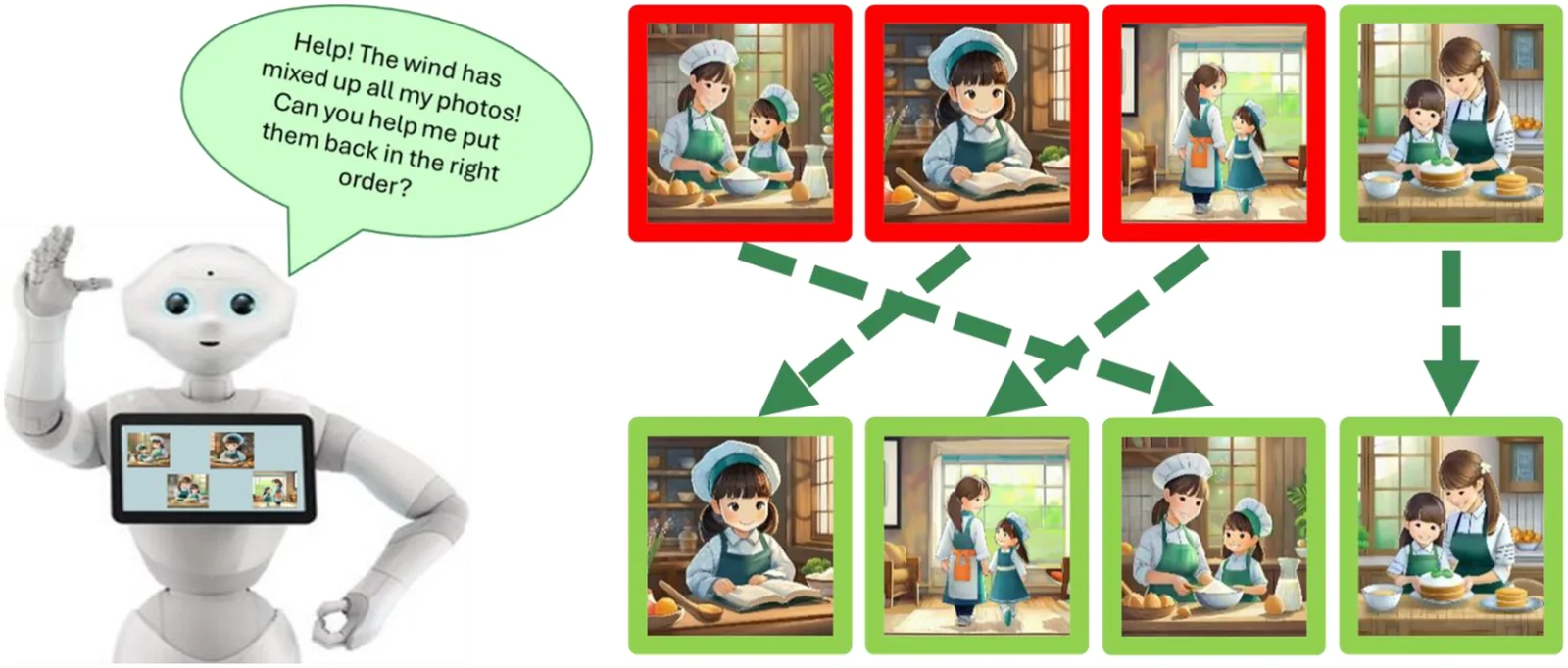
Example of a story.

The picture-based stories training progression will be managed by a custom algorithm, which adapts the sequence of trials according to the participant’s performance. The progressive adaptation of task difficulty and the use of structured interactive challenges are consistent with principles described in gamified learning environments, where adaptive progression and problem-solving activities may contribute to maintaining engagement and cognitive involvement (46). In case of difficulties, facilitations will be provided in order to promote reflection and understanding of the errors made (Figure 3). A narrative framework was also implemented to enhance the child’s motivation and engagement throughout the activity.

**Figure 3:**
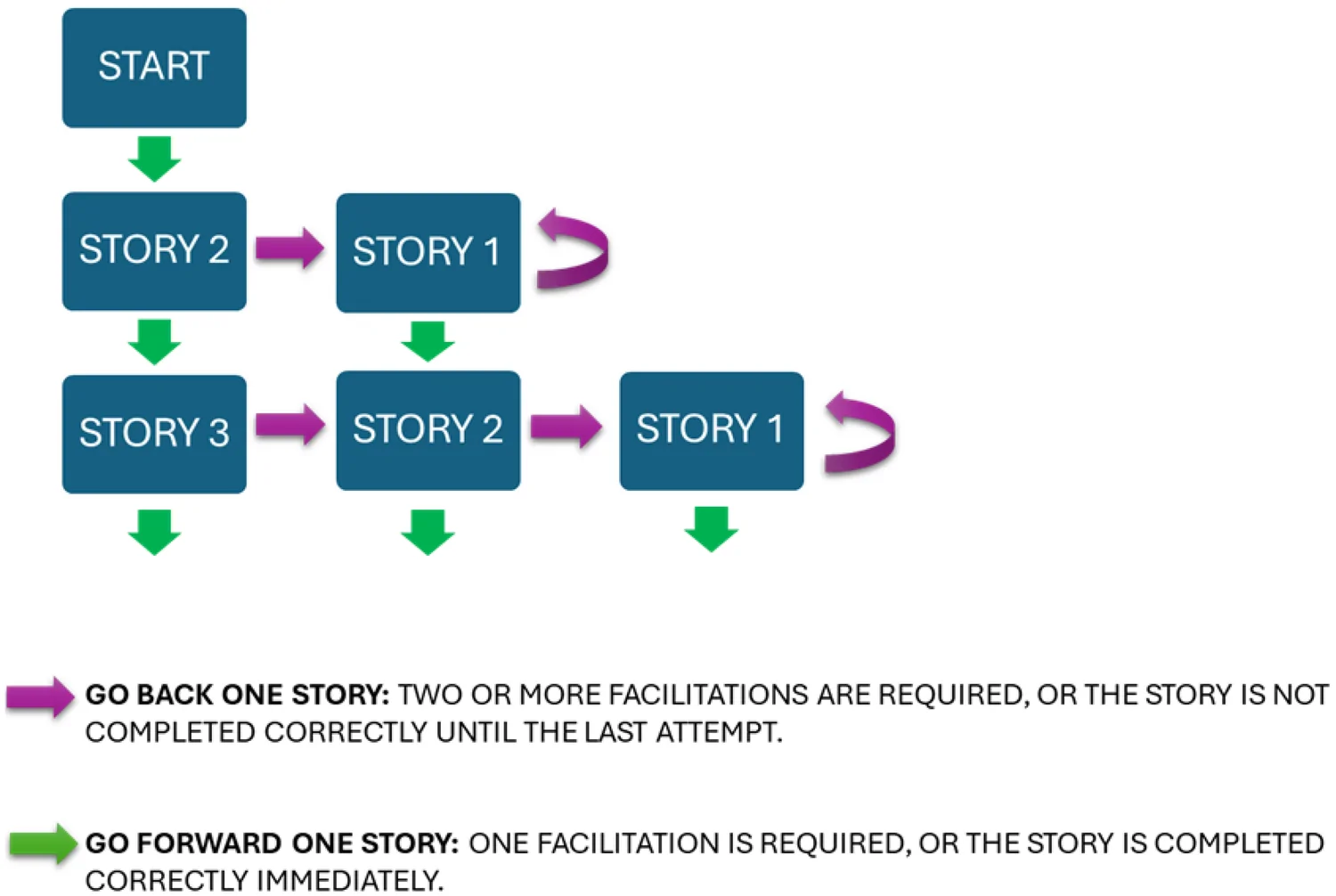
Progression of the session phases.

Each child will participate in approximately 20–25 sessions, delivered 2–3 times per week, with the presence of a clinician trained by the team of engineers at DIEF for the use of the social robot, implemented across the three participating centers. Each session will last approximately 60 min and will follow a standardized structure across centers. To ensure intervention fidelity across participating centers, all clinicians involved in the intervention will receive standardized training on the study protocol, the administration of the training task, and the use of the social robot. A predefined intervention manual will guide session procedures, task administration, and progression rules. Regular coordination meetings will be conducted among participating centers to monitor protocol adherence, discuss potential discrepancies, and ensure consistency in intervention delivery. Before starting the interaction, participants will be also required to wear a wearable sensor on the dominant wrist (e.g., FitBit, USA) to record physiological measures related to physiological activation (e.g., heart rate). During each session, the interaction will be recorded using a camera (Orbbec Camera, USA) and the video will be off-line analyzed to measure interaction parameters. These measures will be aggregated to state the child engagement levels, as detailed in the subsequent section.

### 2.5 Feasibility trial

The feasibility trial has been developed as a preliminary feasibility assessment to evaluate the practicability, usability, acceptability, and engagement level of the task, which was specifically designed for the purposes of this study. The feasibility assessment will be conducted before randomization in all eligible participants and is not intended to evaluate treatment effectiveness. The feasibility assessment will be conducted before randomization in all eligible participants enrolled during the feasibility phase. Up to 100 children are expected to complete this preliminary assessment. Following the feasibility assessment, participants who are considered able to participate in and perform the proposed task, and whose families agree to participate in the study, will proceed to the randomized controlled trial. These criteria will be assessed before randomization and will not determine allocation to the Experimental or Control Group. Eligible participants will then undergo baseline assessment and randomization.

To ensure consistency with the training phase, the feasibility trial will be conducted closely simulating a treatment session. During the session, the participant will be required to sequence picture-based stories starting from an initial level of three images. Task difficulty will be adapted based on performance according to progressive logic: if the child correctly completes the story without any error, they will advance to the next level. In the event of an error, immediate facilitation will be provided by demonstrating the correct placement of the first tile, after which the child will be asked to reorder the same story until completion. If additional errors occur, further facilitation will be provided by progressively adding correctly positioned pictures to the sequence. Upon correct completion of the story, level progression will depend on the number of errors: a single error results in administration of another story at the same level, whereas if more than one error occurs, the subsequent trial will be presented at the preceding level of difficulty. The same procedure is applied to all subsequent stories.

In this way, the child will complete approximately 6 stories, unless they request to stop earlier. The child will be asked to wear the FitBit on the wrist during all feasibility sessions, and the interaction with the robot will be video recorded.

At the end of the feasibility trial, each child will complete a 5-point Likert scale *ad hoc* questionnaire designed to assess acceptability (perceived usefulness, ease of use, user attitude toward the system), usability (efficiency, efficacy and overall satisfaction) and child’s perception of the robot. A similar questionnaire focusing on the proposed activity and its implementation will also be completed by the clinician who administered the task. A mean score of ≥3 on the clinician-rated feasibility questionnaire will be considered indicative of sufficient feasibility to proceed to the randomized phase, consistent with the criterion adopted in previous feasibility studies conducted by our group (47–49). Both questionnaires were developed in accordance with standard definitions of acceptability (50) (51) and usability (52–54) reported in the literature.

At the end of the session, the clinician will be also asked to complete an observational checklist (55) aimed at assessing the child’s engagement during the session, including any tendency to appear frightened or anxious, the difficulty experienced in completing the task, and the level of enjoyment displayed, among other domains. The information obtained from the feasibility assessment will provide evidence regarding the feasibility of the intervention and will support the interpretation of the main trial findings. Feasibility outcomes will be reported separately from the main RCT outcomes and will not be included in the effectiveness analyses.

### 2.6 Social robot platforms and technical setup

Two different humanoid social robots manufactured by SoftBank Robotics, United Robotics Group (France) with similar appearance and software portability will be used in the proposed investigation. Both robots are programmable using Choreographe, the software provided by the manufacturer, and Python 2. Although two different robotic platforms will be used across centers, the intervention protocol, task structure, session duration, and therapist-mediated procedures will be standardized. Robot type will be considered as a potential covariate in statistical analyses.

The Pepper robot is 120 cm tall and made of white plastic, with a soft and friendly design that resembles a human being. It is equipped with 20 motors allowing smooth movements, including arm, head, and mobile base motions. The touchscreen tablet on its chest enables the display of visual content to highlight messages and support verbal communication; users can also interact directly through this interface.

The NAO robot is 57.4 cm tall and, like Pepper, is made of white plastic. Its facial design and overall appearance are similar to Pepper’s. NAO has 25 degrees of freedom, allowing smooth movements of the arms, head, and mobile base. Unlike Pepper, NAO does not have a built-in tablet; therefore, an external tablet of comparable size to Pepper’s will be used.

Both Pepper and NAO are equipped with sensors, cameras, and microphones to detect external stimuli and interact with the surrounding environment. To carry out the study scenarios, *ad hoc* routines replicating the described procedures and two custom user interfaces (one for the children and one for the therapist) will be developed by the University of Florence. The robot is connected to a computer and the routines will be launched at the start of each session. The user interface was developed to show the picture-based stories allowing the child to reorder them; it will be displayed on the robot tablet (in case of Pepper) and on an external tablet (in case of NAO). The therapist interface will be displayed on the computer.

The interaction between the robot and the child will always be supervised by an adult, as specified in the manual. To minimize residual risk, all operators involved in the experimental phase will receive training on the correct use of the device. In addition to the robot, a wearable sensor and a camera for scene recording will also be used. Both devices will be employed according to their intended use. The data collected will be analyzed offline.

### 2.7 Standard care

For ethical reasons, it is not possible to interrupt the children’s usual care, and thus the participation in this project is not intended to interfere with ongoing therapies. Therefore, children assigned both to the EG and CG will continue their usual activities and therapies (standard care) throughout the study.

### 2.8 Outcome measures

Participants of both groups will undergo multiple standardized tests commonly used in clinical practice, including motor and neuropsychological tests and questionnaires, with a total assessment time of approximately two hours. All tests will be administered at all time points, unless otherwise specified. To ensure consistency across centers, all clinicians involved in the intervention will receive standardized training on the study protocol. Regular coordination meetings will be conducted to monitor protocol adherence, discuss potential discrepancies, and ensure consistency in intervention delivery and assessment procedures. Due to the nature of the intervention, complete assessor blinding will not be feasible for all outcome measures that require direct interaction with participants or clinicians involved in the intervention. Standardized assessment procedures and predefined scoring criteria will be adopted across centers to minimize potential assessment bias. Additionally, data collected from devices such as wearable sensors and video cameras will be analyzed using algorithms to assess children’s engagement and interaction with the social robot, alongside *ad hoc* questionnaires and checklists.

#### 2.8.1 Primary pre-post outcome measures

- Picture Arrangement subtest of WISC-III (56). This task assesses nonverbal reasoning, sequential organization, and understanding of cause–effect relationships in social situations. The children are required to rearrange a set of pictures into a logical and coherent sequence that conveys a meaningful story.
- Ad hoc Motor Planning Test. An *ad hoc* task developed by IRCCS Fondazione Stella Maris to assess the ability to reproduce goal-directed motor action sequences. Children are asked to imitate short sequences of actions presented through different model modalities. Performance is scored in terms of execution accuracy and sequential accuracy. The task is administered with the child seated to ensure inclusivity and uniform testing conditions. Preliminary analyses of inter-rater agreement are currently being conducted to evaluate scoring reliability among assessors.

#### 2.8.2 Secondary pre-post outcome measures

##### 2.8.2.1 Standardized test

- Corsi block-tapping subtest (BVS Corsi (57),. This test is used to assess visuo-spatial short-memory and working memory. The child is required to reproduce sequences of blocks previously demonstrated by the examiner by tapping them with the preferred finger, either in the same order (forward condition) or in the reverse order (backward condition). Task difficulty increases progressively with sequence length; the span corresponds to the maximum sequence length correctly reproduced.
- Tower of London test (58) (59). This test is a standardized measure of executive functioning, primarily assessing planning, problem-solving, and goal-directed behavior. The task requires the participant to move colored balls across pegs of different heights to reproduce a target configuration while adhering to specific rules and minimizing the number of moves. Performance indices include accuracy, number of moves, and solution time.
- Leiter-3—Attention Sustained subtest (60) (61). This task assesses the ability to maintain focused attention over time during a nonverbal task. The examinee is required to identify and select target stimuli embedded in distractor matrices of increasing complexity, under time constraints. Performance is indexed by accuracy and error rates.
- NEPSY II—Inhibition subtest (62) (63). The task assesses inhibitory control and cognitive flexibility across three conditions: naming, inhibition of an automatic response, and switching between response rules. In the naming condition, the child is required to name visual stimuli. In the inhibition condition, the child must inhibit the automatic response by providing an incongruent verbal response (e.g., saying “circle” when a square is presented, and “square” when a circle is presented). In the switching condition, cognitive flexibility is assessed by requiring the child to vary the response according to stimulus color. Accuracy and response speed are recorded for each condition.
- NEPSY II—Word Generation subtest (62) (63);. The subtest evaluates lexical access by generating as many words as possible within one minute from a given category (animals, food and drinks) or initial letter (F and S). The number of words produced is recorded for each category.
- NEPSY II—Animal Sorting subtest (62) (63). This subtest evaluates categorization and cognitive flexibility by requiring the child to sort cards into two groups of four, using self-generated criteria and switching between categories as needed.
- TeleFE—Go/No-Go subtest (Anastasis Cooperativa, Project RiDi Fisr2020). The subtest of the TeleFE battery assesses inhibitory control and response monitoring within an executive functioning framework. The participant is required to produce a motor response to target stimuli (Go) and to withhold responses to non-target stimuli (No-Go). Performance is measured through accuracy indices, including commission and omission errors.
- Text comprehension test (Prove MT-3, MT Avanzate and MT 16–19 (64–66). The task assesses reading comprehension skills in children and adolescents. Participants read texts selected according to their grade level, with varying length and complexity levels, and answer questions evaluating literal, inferential, and integrative understanding. Performance is measured by the number of correct responses, reflecting the ability to extract, organize, and integrate information from written text, as well as infer meaning beyond explicit content.
- Invent a story task. In this study, the examiners administered the Invent a Story task, in which children will be asked to orally produce a freely invented story on any theme, with no constraints on length or topic. Children will be explicitly told that their story would not be evaluated, ensuring free expression. Narrative productions will be analyzed for narrative complexity and psychological lexicon usage. Narrative complexity will be coded using the five-level hierarchical system developed by Pinto, Tarchi, and Bigozzi (67), based on the presence or absence of eight structural elements: title, conventionalized opening, identifiable characters, spatio-temporal setting, explicit problem, central event, resolution, and conventionalized closing. Psychological lexicon will be coded according to Tarchi (68), capturing terms related to perceptual states, positive and negative emotional states, volitional expressions, cognitive processes, moral reasoning, and socio-relational dynamics.
- TVPS–4 (Test of Visual-Perceptual Skills, 4th Edition) (69). The test evaluates visual-perceptual abilities, identifying individual strengths and weaknesses across multiple domains, including visual discrimination, visual memory, spatial relationships, form constancy, sequential memory, figure–ground, and visual closure. Performance is measured through accuracy on subtests, providing a comprehensive profile of visual-perceptual functioning independent of motor skills.

##### 2.8.2.2 Questionnaires

- BRIEF-2—parent version (Behavior Rating Inventory of Executive Function, 2nd Edition (70),. This standardized questionnaire is completed by parents or caregivers to assess executive functioning in everyday contexts. Different domains such as inhibition, self-monitoring, flexibility, emotional control, working memory, planning, and organization are considered. Scores provide indices of executive functioning behaviours in daily life, highlighting strengths and difficulties in regulatory and cognitive processes.
- DCD Daily Questionnaire (71) (72). This standardized parent- or caregiver-reported measure assesses the impact of developmental coordination disorder (DCD) on daily activities. Evaluating motor coordination difficulties across various everyday tasks, the questionnaire provides scores that reflect the severity of functional impairment and the child’s motor-related challenges in daily life.
- Abilhand/Abilhand kids questionnaire (73) (74). The ABILHAND (adults) and ABILHAND-Kids (children) questionnaires are standardized self- or proxy-reported instruments assessing manual ability in daily activities. Respondents rate the perceived difficulty of performing various bimanual tasks, providing an index of functional hand use.
- Ad hoc questionnaires assessing the feasibility of the full training. These measures will be created to assess the usability and acceptability of the intervention according to standard definitions reported in the literature (50–54). Both questionnaires will start with an introductory section with open and multiple-choice questions to collect information about the patient, the training just concluded and the robot used. Feasibility questions will be then presented with responses ranked on a 5-point Likert scale (1 = most negative, 5 = most positive). The child version will be adapted to be easily understood, incorporating a smiley meter with five faces representing different expressions, from saddest to happiest, to help the children indicate their responses. Both questionnaires also include open-ended questions, allowing clinicians and children to clarify or expand on their multiple-choice answers, when necessary. The questionnaires will be completed at the end of the training by the referring clinicians and by participants with intellectual and neuropsychological functioning allowing full comprehension of the questions.

#### 2.8.3 Engagement evaluation

During the child’s interaction with the social robot, three different levels of engagement will be measured using different tools (wearable sensors, cameras, microphones). Engagement measures will be calculated as average value computed at the end of the interaction and across different time windows to estimate the engagement dynamics. The framework is based on a preliminary work published in Fiorini (75) and includes:

- **Emotional engagement**, referring to the emotions experienced by the child during the task, will be measured through physiological indicators of heart rate collected via wearable sensors (e.g., Fitbit). In particular, the measurement is expressed as a ratio relative to the reference value obtained from the signal during the three minutes preceding the start of the interaction. Additionally, OpenFace tool (76) will be used to estimate the most prevalent emotions occurred during the interaction using the Action Units (AUs).
- **Cognitive engagement**, defined in terms of attention span and cognitive load during task execution, will be assessed through eye-gazing metrics extracted offline from video recordings. These metrics will be computed through tools such as DeepGaze or Gaze360 (77), which enable the estimation of the proportion of time the child’s attention is directed to task-relevant versus non-relevant stimuli, as well as the calculation of gaze entropy. The cognitive load will be derived from the blinking rate (OpenFace, AU) (78). Keypoints related to the eyebrow dynamics and head pose will also be tracked over time to corroborate measures related to the child attention during the task. Cases where the child requires assistance from caregivers will be also recorded.
- **Behavioral engagement** will be measured through the analysis of the game log to estimate the task performance. Variables considered include the number of times that the verbal description of the story or individual frames was replayed, the number and type of errors (e.g., sequencing errors), and the total duration of the task together with the duration for each specific story. In addition, OpenSmile software (79) will be used to analyze the audio recordings of the interaction.

Custom observational grids will be also provided to the therapist to observe the interaction and keep track of the events that may occur. Additionally, at the end of interaction the therapist will be also requested to fill in a brief scale to evaluate the perceived engagement, inspired by the questionnaire developed by Martinovic (55), during each training session.

### 2.9 Statistical analysis

Data will be analyzed using specialized software (e.g., SPSS v21, Python, and MATLAB 2024b). Descriptive statistics will be used to summarize demographic and clinical characteristics of the sample and to assess the distributional properties of the outcome variables. Baseline characteristics will be compared between groups to evaluate the comparability of the Experimental Group (EG) and Control Group (CG).

The primary hypothesis is that children receiving the social robot-mediated Learning by Teaching intervention will show greater improvements in planning abilities compared with children receiving standard care. The primary outcomes will include changes in planning abilities assessed through the Picture Arrangement subtest of the WISC-III and the *ad hoc* motor planning task from baseline (T0) to post-intervention assessment (T1). Follow-up data collected at T2 will be used to examine the maintenance of intervention effects in the Experimental Group and post-treatment changes in the Control Group following the intervention.

The primary analysis will be conducted according to the intention-to-treat (ITT) principle. The ITT population will include all participants allocated to the Experimental Group or Control Group and will be analyzed according to their original group allocation, irrespective of adherence to the intervention or subsequent protocol deviations. All available repeated outcome measurements from ITT participants will be included in the linear mixed-effects models. A per-protocol (PP) analysis will be conducted as a secondary sensitivity analysis. The PP population will include participants who complete at least 80% of the scheduled intervention sessions in the Experimental Group, undergo the post-intervention assessment at T1, and have no major protocol deviations likely to affect the evaluation of the intervention effect. For the Control Group, participants will be included in the PP population if they undergo the scheduled assessment at T1 and have no major protocol deviations. As standard care consists of the continuation of each child’s usual activities and therapies, without a predefined number or frequency of sessions within the study protocol, adherence to standard care will not be quantified using the 80% criterion.

Repeated measurements collected at T0, T1, and T2 will be analyzed using linear mixed-effects models. The models will include fixed effects for group, time, and the group-by-time interaction, together with an appropriate participant-level random effect to account for within-participant correlation. The primary treatment effect will be estimated through a prespecified contrast of the group-by-time interaction representing the between-group difference in change from T0 to T1. Changes from T1 to T2 will be examined separately within each group to evaluate the maintenance of intervention effects in the Experimental Group and the post-treatment changes in the Control Group following the intervention. Study center, robot platform (Pepper vs NAO), age, and diagnostic category will be included as prespecified covariates, provided that their inclusion is supported by the distribution of the data and model identifiability. Within-group changes will be reported only as secondary descriptive analyses. For all main comparisons, effect estimates, standardized effect sizes, 95% confidence intervals, and exact *p*-values will be reported.

Secondary outcomes will include executive functions, visuospatial memory, attention, inhibition, narrative production, visual-perceptual abilities, reading comprehension, and functional measures assessed using standardized tests and questionnaires. Analyses of these outcomes will examine the possible generalization of intervention effects beyond the specifically trained planning abilities. The Holm-Bonferroni procedure will be applied to control for multiplicity across the primary outcome measures. Secondary-outcome analyses will be interpreted as exploratory and will also be adjusted for multiple comparisons using the Holm-Bonferroni procedure. Missing data patterns and dropout characteristics will be examined and reported. Linear mixed-effects models will include all available repeated measurements from participants in the ITT population under the assumption that data are missing at random conditional on the variables included in the model. Multimodal engagement measures derived from wearable sensors, video, and audio recordings will be considered mechanistic variables reflecting cognitive, emotional, and behavioral involvement during the interaction. Associations between engagement features and changes in cognitive, motor, and functional outcomes will be investigated using correlation and regression analyses. Furthermore, exploratory mediation analyses will be conducted to examine whether engagement contributes to the relationship between intervention participation and improvements in cognitive, motor, and functional outcomes. Since the study was not specifically powered to detect mediation effects, these analyses will be considered exploratory.

## 3 Discussion

To our knowledge, this is the first study aiming to explore the effectiveness of an innovative, structured intervention based on the Learning by Teaching paradigm, mediated by a social robot, for children with neuromotor impairments. Unlike many existing studies that focus on single-session interactions or short-term feasibility, the proposed approach is conceived as a multi-session intervention, designed to be delivered repeatedly over time for each child. This longitudinal structure allows the intervention to target learning, engagement, and rehabilitation processes more consistently, addressing a gap in the current literature, as few studies have investigated the sustained effects of robot-mediated interventions in this population.

The main novelty of this intervention lies in the use of the robot as a mediating tool that not only stimulates cognitive functions but also promotes emotional and metacognitive processes through social interaction (9).

A central element of the intervention is the child’s active role as the “teacher,” which, through the Learning by Teaching paradigm, fosters the acquisition of metacognitive skills and the ability to take another’s perspective. This paradigm has been shown to improve not only children’s understanding of the material but also their motivation and self-efficacy (19) (20). This distinctive feature could potentially enhance intervention effectiveness, resulting in potential benefits not only in terms of behavioral engagement, but also in higher-order cognitive functions. The training also allows for simultaneous improvement of both cognitive and motor domains, by using a picture sequencing task. In fact, in addition to logical reasoning and higher-order cognitive skills, including EF components, this training also engages motor planning processes, supporting anticipatory control, temporal organization of actions, and motor programming. This integrative approach provides a diverse and personalized intervention tailored to each child’s abilities and may enhance not only targeted skills but also functional abilities relevant to daily life, such as reading comprehension, problem-solving and the execution of goal-directed actions—by strengthening the underlying cognitive and motor processes trained. Another strength of the study is the use of a multimodal framework based on sensors to assess engagement, integrating cognitive, behavioral, and physiological indicators. This comprehensive assessment will provide a nuanced understanding of the child’s interaction with the robot, highlighting individual differences in engagement and responsiveness (3). Indeed, we are moving toward real-world applications, so it is difficult to rely only on observed data. It is imperative to determine a minimum set of features that can measure child-robot interaction in an efficient way. Importantly, these measures will also be used to examine the predictive role of engagement in the effectiveness of the intervention, allowing us to determine how variations in emotional, cognitive, and behavioral involvement may influence improvements in cognitive, motor, and functional outcomes. Insights from this approach may inform the optimization of the intervention to better tailor it to each child’s specific needs and maximize its impact.

Despite these strengths, certain limitations should be acknowledged. The structured clinical setting may not fully reflect daily life environments, potentially limiting the generalizability of the findings. Additionally, variability in age and developmental stage among participants could influence both the interaction with the robot and the observed outcomes, requiring careful consideration in data interpretation. Furthermore, complete blinding and random assignment will not be feasible for all assessments, representing a potential limitation of the study. Allocation will not be concealed, which may introduce a risk of selection bias; this limitation will be considered when interpreting the findings of the trial. The absence of an active technology-matched control condition also limits the possibility of fully isolating the specific contribution of the robot’s social presence from the effects of the task itself. Potential adverse or unintended effects associated with the intervention will also be considered, including fatigue, frustration, anxiety, or discomfort during interaction with the social robot, as well as possible novelty and expectancy effects. Given the novelty of the intervention and the limited availability of directly comparable evidence, the sample size was based on related interventions and outcomes. The findings may therefore inform future studies with more precise sample size estimations. In addition, given the novelty and specific focus of the study, the *ad hoc* motor-planning measure was developed specifically for this protocol, and its inter-rater reliability has not yet been established, representing a further limitation. These aspects will be monitored by the supervising clinician throughout the sessions. In addition, technical issues related to wearable sensors and video/audio recordings may occur and will be managed according to predefined procedures. Video and audio data will be collected exclusively for research purposes and handled according to data protection procedures approved by the Ethics Committee. Finally, the use of two different robotic platforms (Pepper and NAO) may introduce variability in the interaction experience; however, standardized intervention procedures and statistical control of robot type will be adopted to minimize this potential confounding effect.

In conclusion, this study could contribute significantly to understanding how social robots can support cognitive and motor rehabilitation in children with neuromotor impairments. The findings may also guide future studies exploring long-term effects and applicability in ecological contexts, such as schools or homes, to assess transferability and sustainability of the intervention in everyday life.
